# Animal Models of Zika Virus Infection during Pregnancy

**DOI:** 10.3390/v10110598

**Published:** 2018-10-31

**Authors:** Elizabeth A. Caine, Brett W. Jagger, Michael S. Diamond

**Affiliations:** 1 Department of Medicine, Washington University School of Medicine, Saint Louis, MO 63110, USA; ecaine@wustl.edu (E.A.C.); bjagger@wustl.edu (B.W.J.); 2 Department of Pathology & Immunology, Washington University School of Medicine, Saint Louis, MO 63110, USA; 3 Department of Molecular Microbiology, Washington University School of Medicine, Saint Louis, MO 63110, USA; 4 The Andrew M. and Jane M. Bursky Center for Human Immunology and Immunotherapy Programs, Washington University School of Medicine, Saint Louis, MO 63110, USA

**Keywords:** Zika virus, vaccines, animal models, pregnancy, non-human primates, mice, congenital Zika syndrome, pathogenesis

## Abstract

Zika virus (ZIKV) emerged suddenly in the Americas in 2015 and was associated with a widespread outbreak of microcephaly and other severe congenital abnormalities in infants born to mothers infected during pregnancy. Vertical transmission of ZIKV in humans was confirmed when viral RNA was detected in fetal and placental tissues, and this outcome has been recapitulated experimentally in animals. Unlike other flaviviruses, ZIKV is both arthropod- and sexually-transmitted, and has a broad tissue tropism in humans, including multiple tissues of the reproductive tract. The threats posed by ZIKV have prompted the development of multiple in vivo models to better understand the pathogenesis of ZIKV, particularly during pregnancy. Here, we review the progress on animal models of ZIKV infection during pregnancy. These studies have generated a foundation of insights into the biology of ZIKV, and provide a means for evaluating vaccines and therapeutics.

## 1. Introduction

Zika virus (ZIKV) is a mosquito-transmitted, positive-sense RNA virus in the *Flaviviridae* family. ZIKV was first isolated from a rhesus macaque in the Zika Forest of Uganda in 1947 [1]. Historically, human cases were rarely reported, with ZIKV infections being clinically unapparent, causing only mild fever, malaise, and a rash, or being misdiagnosed as Dengue virus infections. However, beginning in 2007 in the Yap Islands of Micronesia [2] and subsequently in 2013 in French Polynesia [3], the epidemiology of ZIKV changed, with higher rates of symptomatic disease, including an association with Guillain-Barré syndrome in adults, and evidence of epidemic transmission [4]. ZIKV emerged in the Western Hemisphere in 2015 and caused epidemics in Central and South America and the Caribbean islands [5]. During its spread throughout the Americas, ZIKV became a public health concern due to its ability to cross the placenta and infect neuroprogenitor cells in the fetal brain, leading to microcephaly, congenital abnormalities, preterm birth, and death [6].

Thousands of infants in the Americas have been born with congenital ZIKV syndrome (CZS), which will impair their future neurodevelopmental function [7]. CZS is defined by severe microcephaly; damage to the eye, including chorioretinal atrophy; and congenital muscular contractures that restrict body movement [8]. Although microcephaly has been the hallmark of CZS, many infants exposed to ZIKV in utero are born without this morphological anomaly. However, infants without microcephaly have been diagnosed with epilepsy, hypertonia, and a decreased brain volume [9]. On a microscopic level, ZIKV infected fetuses showed evidence of the altered migration of neuroprogenitor cells, neuronal apoptosis, and lesions in gray matter structures.

A correlation has been seen between the trimester of exposure to ZIKV and fetal outcome. In one study in Brazil, 55% of first trimester infections resulted in fetal abnormalities compared to only 29% during the third trimester [6]. In the United States, maternal ZIKV diagnoses during the first, second, or third trimester resulted in 6%, 5%, or 4% rates of ZIKV-associated birth defects, respectively [10]. Another cohort in the United States reported that 11% of first trimester ZIKV exposures resulted in CZS, whereas no complications were seen in completed pregnancies that were ZIKV exposed in the second and third trimesters [11]. One woman in her 11th week of gestation (first trimester) was diagnosed with persistent viremia (10 weeks after the onset of clinical symptoms) that resulted in fetal brain abnormalities, including decreased neuronal white matter and subventricular zone volume [12]. It remains unclear whether environmental or immunological co-factors have contributed to the higher rates of ZIKV-induced congenital anomalies seen in Brazil compared to other parts of the Americas.

In response to the ZIKV pandemic and the outbreak of congenital disease, animal models were developed to study ZIKV pathogenesis and evaluate countermeasures, including new vaccines and therapies. The ability to control the infection route and gestational age has allowed researchers to confirm the teratogenic effects of ZIKV infection and study both host and viral factors associated with vertical transmission and neuropathogenesis.

## 2. Mouse Models

Mice are the most commonly used animals to model viral infections in humans. Their low cost, short breeding times, large litters, and ease of handling facilitate their widespread use. They often recapitulate important aspects of human disease, including after viral and bacterial infections [13,14,15]. Mice have also been used widely to evaluate correlates of protection for vaccines and therapeutics before the initiation of clinical studies in human subjects [16,17]. Moreover, the ability to manipulate mice genetically (transgenes, CRISPR-Cas9 mutations, homologous recombination, and conditional deletions) to examine how particular genes influence infection and immunity makes them a preferred animal for many in vivo studies.

In the context of pregnancy, there are advantages and disadvantages for using mice to study human infection and disease. Specific breeding schemes with knockout mice allow for the study of the function of individual genes by restricting their expression to the maternal or fetal side of the placenta. However, mice have a much shorter gestation period compared to humans, only 20 days, and the structure of their placenta differs [18]. Even so, when Brazil saw an increase in microcephaly cases in 2015 associated with the outbreak of ZIKV, the high throughput and availability of mouse infection platforms during pregnancy allowed for the first direct causal link between ZIKV and fetal abnormalities to be established [19].

### 2.1. Type I Interferon Receptor Deficient Mouse Models

Peripheral challenge of adult wild-type (WT) C57BL/6 mice with ZIKV resulted in little virus replication and no disease [20]. One reason for the resistance of WT mice to peripheral infection is that ZIKV fails to antagonize mouse Stat2, a key intermediate in the IFN signaling pathway, whereas it can promote degradation of human STAT2 [21,22]. To dampen the innate immune response and facilitate ZIKV replication in mice, the initial mouse models in the field utilized type I interferon (IFN) receptor knockout mice (*Ifnar1*^−/−^) or administered a blocking anti-type I IFN receptor antibody (MAR1-5A3) (Table 1). For vertical transmission studies, two models were developed: WT C57BL/6 females were crossed to WT C57BL/6 sires, and then dams were given the anti-Ifnar1 antibody immediately before ZIKV infection; or *Ifnar1*^−/−^ dams were crossed to WT sires and then inoculated with ZIKV. Subcutaneous infections with ZIKV (French Polynesia strain) in these models on embryo day (E)6.5 and harvested seven days later on E13.5 showed the vertical transmission of ZIKV to the placenta and fetal heads [19]. Decreased fetal head and body size and increased resorption rate were seen in fetuses from ZIKV-infected *Ifnar1*^−/−^ dams. Intravaginal inoculation of *Ifnar1*^−/−^ dams mated to WT C57BL/6 sires at E4.5 and E8.5 also led to fetal resorption and viral replication in the placenta and fetus on E18.5 [23]. Examination of the placenta revealed that ZIKV infected trophoblasts and endothelial cells, which triggered apoptosis, vascular damage, and placental insufficiency. In these infection models, dams sustained severe ZIKV infection, as evidenced by high levels of viral RNA in the serum, spleen, and brain [19,23].

The effects of type III IFNs during ZIKV infection have been evaluated in pregnancy using *Ifnlr1*^−/−^ dams crossed to WT C57BL/6 or *Ifnlr1*^−/−^ sires. *Ifnlr1*^−/−^ dams were treated with an anti-Ifnar1 antibody and then inoculated with ZIKV on E12. Whereas *Ifnlr1*^−/−^ × *Ifnlr1*^−/−^ matings resulted in an increase in viral infection in the placenta and fetal heads, *Ifnlr1*^−/−^ dam × WT C57BL/6 sire matings had a similar viral burden to WT C57BL/6 mice. This data suggested that the feto-placental unit had to be fully deficient in type III IFN signaling [46] to be affected, although an isolated deficiency of Ifnlr1 in the fetuses (obtained through an *Ifnlr1*^+/−^ dam × *Ifnlr1*^−/−^ sire cross) was not tested. Treatment with pegylated mouse IFN-λ2 at E12 but not E6 reduced the ZIKV burden in the placenta and fetal heads late in gestation. Subsequent studies showed that the protective effect of IFN-λ2 required a developed placenta, which occurs by E10. These studies established the role of type III IFN signaling in restricting ZIKV infection in the placenta in a gestational stage-dependent manner, and have been corroborated in human tissues [46,54].

### 2.2. Immunocompetent Mouse Models

ZIKV infection in non-pregnant and pregnant immunocompetent C57BL/6, BALB/c, Swiss Webster, or CD-1 mice (via subcutaneous, intraperitoneal, or intravenous routes) resulted in no clinical signs of disease and little viral replication [20,55], presumably because infection was aborted in the setting of intact innate immunity. Nonetheless, closer analysis revealed that some viral replication does happen in these mice, and that in certain circumstances, vertical transmission and accompanying fetal abnormalities can occur. WT C57BL/6 dams inoculated via an intravaginal route with ZIKV shed viral RNA in vaginal washes for six days [23]. Viral particles were reported in the cortex and cerebellum of brains isolated from these fetuses by electron microscopy, even though, paradoxically, viral RNA was not detected by real-time RT-PCR [23]. Intraperitoneal inoculation of pregnant WT C57BL/6 dams led to the infection of radial glia cells of the dorsal ventricular zone of the infected fetuses [28]. ZIKV injected directly into the amniotic fluid or uterus of pregnant WT C57BL/6 mice at E13 or E15 resulted in spontaneous abortions, spinal cord defects, and motor neuron and ocular malformations or intracranial calcifications in surviving mice [42,47]. WT SJL mice that were inoculated via an intravenous route with a very high dose of a Brazilian strain of ZIKV developed intrauterine growth restriction (IUGR) and microcephaly [31].

Fetal abnormalities in these WT mouse models were only seen when non-physiological routes of infection were used, including intrauterine, intravenous, or direct injection into the amniotic fluid or uterus. In an effort to create a peripheral challenge model with a more immunocompetent mouse, one group replaced the mouse *Stat2* gene with the human *STAT2* ortholog (hSTAT2-KI mice) [37]. This substitution allowed ZIKV to antagonize human STAT2 in mice to overcome some of the innate immune restrictions [21]. For these studies, a mouse adapted ZIKV (Dakar strain) with an adaptive amino acid change in the NS4B protein (G18R) was created by passaging in *Rag1*^−/−^ mice, which lack mature B and T cells [37]. Using a combination of the adapted ZIKV Dakar strain with hSTAT2-KI mice, a model of vertical transmission was demonstrated after subcutaneous infection. High levels of ZIKV RNA were isolated from the placenta and fetal heads of these mice.

### 2.3. Effects of Type I IFN Signaling on Fetal Resorption

In the context of the studies with type I IFN deficiency, it was found that factors other than a viral burden contribute to the fetal outcome. Type I IFN signaling in the placenta was shown to contribute independently to fetal resorption [56]. *Ifnar1*^−/−^ dams mated to WT C57BL/6 sires yield pups that are heterozygous for Ifnar1 expression. Despite having the ability to respond to type I IFN, these fetuses were not protected from ZIKV, and many were resorbed [19,23]. However, when type I IFN signaling was completely deficient in the fetus (by *Ifnar1*^−/−^ × *Ifnar1*^−/−^ matings), paradoxically, greater survival was observed after intravaginal inoculation on E5.5 than with fetuses that had an intact type I IFN response [56]. Remarkably, the ZIKV burden in the placenta and fetal head was higher in fetuses from *Ifnar1*^−/−^ × *Ifnar1*^−/−^ than *Ifnar1*^−/−^ × WT C57BL/6 matings, such that the viral load did not directly correlate with the outcome. Type I IFN signaling in the placenta was shown to instigate fetal demise and may serve as a way to select against the development of fetuses that have been exposed to and sustained significant viral infections or inflammation. The effect of intact type I IFN signaling on fetal resorption was observed when WT C57BL/6 dams mated with WT C57BL/6 sires were inoculated intravenously with a Puerto Rican strain of ZIKV at E9.5. These fetuses were resorbed at day E17.5, even in the absence of viral RNA in fetal heads [35].

### 2.4. Gestational Stage Effects

More severe fetal abnormalities correlate with the trimester in which a pregnant human is infected with ZIKV [57]. In a large cohort of 301 confirmed ZIKV infections during pregnancy from French Guiana, mothers infected during the first and second trimesters had a greater risk of severe fetal abnormalities, compared to infections during the third trimester [58]. Consistent with this data, pregnant CD1 mice inoculated via intrauterine injection with ZIKV had greater fetal viability and lower viral RNA levels 48 h later when infected at E14 instead of E10 [36]. Similarly, pregnant C57BL/6 mice inoculated with ZIKV had a greater number of resorbed fetuses five days post infection when they were inoculated at E9.5 compared to E12.5 [35]. Moreover, *Ifnar1*^−/−^ dams mated to WT C57BL/6 sires had fetuses with reduced head circumferences and crown rump length when infected with ZIKV at E9 but not E12 [46]. Viral burden and the rates of fetal demise were highest in mice infected at E6, compared to E9 and E12. Finally, growth defects were observed in pups of Swiss-Webster dams inoculated with ZIKV at E4 and E8, but not E12 [43].

### 2.5. Summary

Mouse models provided a rapid platform for confirming the teratogenic potential of ZIKV infection. Fetal abnormalities were observed in fetuses from ZIKV infected pregnant *Ifnar1*^−/−^ mice that were mated to WT sires. Viral RNA was isolated from the placentas and fetal heads, with gestational age being a determinant of increased viral burden. Type I IFN was found to be a determinant of fetal resorption after ZIKV infection. Type III IFN-λ responses protected the placenta from ZIKV, and dams treated with pegylated IFN-λ2 had improved pregnancy outcomes. An immunocompetent hSTAT2-KI model was recently developed and also showed vertical transmission after subcutaneous ZIKV infection. Overall, mice have been used successfully to model many aspects of ZIKV infection during pregnancy and provided a way for establishing correlates of protection for ZIKV countermeasures.

## 3. Development of ZIKV Countermeasures in Pregnant Mice

### 3.1. Vaccines

The epidemic of CZS in the Americas has spurred an accelerated effort by academia, the government, and industry to develop vaccines capable of preventing the potentially devastating consequences of maternal ZIKV exposure for the developing fetus. In the first description of the efficacy of ZIKV immunization against challenge during pregnancy, WT C57BL/6 dams were immunized with either live-attenuated NS1-mutant (LAV) or lipid nanoparticle-encapsulated mRNA (mRNA-LNP) vaccines [24]. Approximately one month after primary immunization or secondary boosting, females were mated with WT C57BL/6 sires. Pregnant dams were treated with an anti-Ifnar1 blocking antibody on E5, followed by subcutaneous challenge with ZIKV on E6. Placental and fetal tissues harvested on E13 revealed wide dissemination of ZIKV in placebo-vaccinated dams, with a high viral burden in the placenta and rates of transmission to fetal heads. In contrast, mice immunized with either the LAV or mRNA-LNP vaccine showed 100,000-fold reductions in placental ZIKV infection, with most free of any detectable ZIKV RNA. The findings in the placenta were recapitulated in the fetal heads, with the majority of both ZIKV vaccine groups showing no evidence of infection. Consistent with these data, fetal viability was markedly greater at term after vaccinating with the mRNA-LNP vaccine, whereas resorption and fetal demise were uniformly seen in placebo-immunized mice. A similar WT dam + anti-Ifnar1 antibody model was employed to evaluate a 3′-untranslated region mutant ZIKV LAV. In these studies, maternal serum neutralization titers inversely correlated with placental viral burden, suggesting a humoral correlate of vaccine protection against CZS [27]. The strengths of this challenge model in pregnant mice include the low cost and ease of scalability owing to the use of commercially-procured WT mice and a transient anti-Ifnar1 blockade. However, the anti-Ifnar1 blockade is an imperfect approximation of ZIKV-mediated type I IFN antagonism, since all cells (not just ZIKV-infected cells) are affected. Indeed, despite serum neutralizing antibody titers of 1:10,000 in some vaccinated mice, ZIKV seeded and infected a proportion of placentas after peripheral subcutaneous challenge, and even transmitted the virus to some fetal heads. However, complete protection was seen in the placenta and fetal heads of fetuses from *Ifnar1*^−/−^ A129 dams that were immunized with a different live attenuated ZIKV vaccine containing deletions in the capsid protein [53].

Subsequent reports of vaccine efficacy in pregnant mice have made modifications that may provide specific advantages. For example, WT C57BL/6 (H-2^b^) females were primed by prior ZIKV infection and mated to allogeneic, WT BALB/c (H-2^d^) males [48]. Allogeneic mating was hypothesized to better mimic the typical situation in human pregnancy, where maternal tolerance of paternal antigens modifies the immunologic state of the placenta, in particular, the accumulation of immunosuppressive regulatory CD4^+^ T cells, to maintain a state of tolerance to the fetus. The dams were given no immunosuppression during priming by ZIKV, and exhibited no signs of illness during this primary infection as a result. However, even this abortive infection was sufficient to protect fetuses after subsequent anti-Ifnar1 antibody treatment and ZIKV challenge during pregnancy (at E10 or E11), as judged by the markedly decreased ZIKV RNA burden in feto-placental units three days after challenge. Consistent with the earlier reports, a sizeable subset of feto-placental units was still positive for ZIKV RNA, despite high titers of circulating neutralizing antibody.

In addition to studying infection and disease in fetuses, some groups have followed pups from vaccinated dams through birth or challenged immediately after birth. ICR female mice were immunized with a recombinant E ectodomain protein (E90: 450 amino acids) vaccine and mated with ICR sires. Pregnant dams were challenged with ZIKV at E13.5 in the cerebral lateral ventricle of the fetal brain. Immunized fetuses inspected five days later were protected from infection and neuroprogenitor cell injury, and neonates from vaccinated dams were protected against microcephaly [44]. The performance of a ZIKV-JEV chimeric LAV (prM-E genes of ZIKV) was evaluated in immunocompetent, four-week-old BALB/c females [49]. Sixty days after immunization, vaccinated dams were challenged at E13 after mating with WT males by intraperitoneal injection. In these experiments, no maternal viremia was detected. In a permutation of this model, one-day-old suckling mice from the vaccinated dams were challenged via intracranial injection. This resulted in universal death of the control pups, but 100% survival of pups born to ZIKV-JEV LA-immunized dams. This approach offers the theoretical advantage of avoiding exogenous immunosuppression, but at the expense of preventing the assessment of vaccine performance against transplacental transmission. As an alternative to a transient type I IFN blockade, in an experiment with a limited number of animals, females deficient in both type I and II IFN receptor signaling (*Ifnar1*^−/−^ *Ifngr1*^−/−^) were utilized to evaluate a codon pair deoptimized LAV ZIKV vaccine [50]. Pregnant *Ifnar1*^−/−^ *Ifngr1*^−/−^ dams were challenged with ZIKV six days after mating to *Ifnar1*^−/−^ *Ifngr1*^−/−^ sires, which resulted in 100% of unvaccinated dams succumbing prior to delivery, whereas all immunized dams gave birth to healthy-appearing pups. However, no further virological, morphological, or behavioral assessment of the pups was reported.

### 3.2. Antibody Treatment

Studies of human patients have suggested roles for humoral immunity in controlling natural ZIKV infection [59]. Based on this observation, several groups have pursued the identification of strongly neutralizing anti-ZIKV antibodies with potential therapeutic application [60]. The highly neutralizing human monoclonal antibody (mAb) ZIKV-117 was evaluated in pregnant mice [26]. *Ifnar1*^−/−^ dams were mated to WT sires, and ZIKV-117 was administered the day prior to ZIKV challenge. Although treatment with an isotype control mAb resulted in fetal demise and resorption, the vast majority of ZIKV-117-treated fetuses remained morphologically intact at E13. The authors also used the WT dams + anti-Ifnar1 mAb model to show that the passive transfer of ZIKV-117 as prophylaxis or post-exposure therapy resulted in markedly reduced placental and fetal viral burdens, although some tissues remained positive for ZIKV RNA. ZIKV-117 treatment also significantly improved placental and fetal dimensions to levels similar to uninfected animals. Introduction of the L234A/L235A “LALA” mutations, which abrogates Fc-FcγR interaction and prevents antibodies from mediating antibody-dependent enhancement, did not alter the efficacy of ZIKV-117 against placental or fetal infection, suggesting that antibody effector functions were not required for the therapeutic efficacy of ZIKV-117.

A similar scheme was utilized to assess the efficacy of the cross-reactive, envelope-dimer epitope (EDE) mAb EDE1-B10 when given to pregnant mice infected with various strains of ZIKV [25]. *Ifnar1*^−/−^ dams were mated to WT sires and challenged subcutaneously on E6 with a Brazilian ZIKV strain, followed by single-dose EDE1-B10 treatment on the day following infection. A nine-fold reduction in the rate of fetal demise was observed in mAb-treated mice, and this correlated with reductions in ZIKV-induced damage to the junctional zone of the placenta. Administration of EDE1-B10 mAb to WT + anti-Ifnar1 mAb-treated mice one day after maternal infection with a mouse adapted ZIKV (Dakar strain) resulted in a markedly reduced ZIKV burden in the placenta (660,0000-fold reduction) and fetal head (4900-fold reduction), with a substantial fraction of placentas and fetal heads containing no detectable virus. Notably, a LALA-mutant of EDE1-B10 performed similarly in the WT + anti-Ifnar1 model. Treatment with EDE1-B10 at a later time point (i.e., three days following infection) in the WT + anti-Ifnar1 model also reduced ZIKV RNA in maternal and fetal tissues, but to a lesser degree, implying that antibody-based therapeutics may need to be administered within a certain time window after infection to convey maximal benefit. This also suggests that antibody-mediated immune clearance of established placental ZIKV infection may be subject to physiologic limitations.

In a separate report, Regla-Nava et al. utilized *Ifnar1*^−/−^ dams mated to WT sires to test the consequences of antecedent DENV infection on secondary ZIKV infection during pregnancy [51]. Fetuses within DENV2-immune dams were noted to maintain a normal size and weight after ZIKV challenge on E7, in contrast to non-immune controls. Anti-CD8^+^ T cell depletion abrogated this protective effect of DENV immunity on the fetus. Anti-CD4^+^ T cell depletion also increased the fetal resorption rate, but only in a subset of animals. The central role of CD8^+^ T cells in mediating cross-immunity against ZIKV during pregnancy was recapitulated by CD8^+^ T cell depletions in the WT dams + anti-Ifnar1 mAb model and in *CD8*^−/−^ dams. Thus, for both humoral and cell-mediated mechanisms, murine models have generated evidence for protective mechanisms that can be corroborated in non-human primate models and ultimately, in humans.

## 4. Non-human Primate (NHP) Models

The non-human primate (NHP) model of ZIKV infection in pregnancy shares greater similarities with humans than mice [61,62]. NHPs have comparable placental architecture to humans and longer gestation times (macaques, 146–186 days) than rodents and ruminants [63,64] and have been used extensively to study the infection and treatment of other TORCH (Toxoplasmosis, Other [syphilis, varicella-zoster, HIV, parvovirus B19], Rubella, Cytomegalovirus (CMV), and Herpesvirus infections) pathogens [65,66,67,68,69]. Among NHPs, macaques (*Macaca*) have been used most frequently for the study of ZIKV pathogenesis during pregnancy (Table 2). The first infection models of pregnancy were generated in pigtail (*Macaca nemestrina*) and rhesus (*Macaca mulatta*) macaques with isolates of ZIKV from French Polynesia, Cambodia, Brazil, and Puerto Rico. Vertical transmission of ZIKV was reported, although fetal abnormalities and brain injury were relatively minor [70,71,72]. However, more recent ZIKV infection studies on pregnant rhesus macaques have demonstrated significant fetal demise using primary isolates from Brazil, as well as larger groups of infected animals [73].

### 4.1. Maternal Syndrome in NHPs

Most ZIKV-infected pregnant pigtail and rhesus macaque dams appeared healthy, which is analogous to the asymptomatic or mildly symptomatic infections of humans [74]. Minor clinical signs were reported that lasted through the first week of infection. In several rhesus and pigtail macaques, maternal tissues at term (brains, spleens, eyes, kidney, liver, lymph node, saliva, and decidua) were positive for ZIKV RNA [70,72]. One consistent observation was the presence of ZIKV RNA in urine in many rhesus macaque dams for up to two weeks after infection [71,72]. Longitudinal sampling studies revealed persistent viral RNA in the serum and plasma in a subset of pregnant NHPs, which has also been reported during human pregnancies [12]. ZIKV RNA was present in the plasma/sera of both pigtail and rhesus macaques for 11 to 70 days post infection, with peak viremia observed within the first week [71,72,73,75,76]. One study observed that NHPs infected with ZIKV Brazil in the first trimester exhibited longer periods of sustained viremia (28 to 70 days) then those inoculated during the second trimester (14–42 days) [75]. Common marmosets (*Callithrix jacchus*) infected with ZIKV also had prolonged viremia and viruria [77], which correlated with the spread to fetal and maternal tissues. These observations are notable since persistent viremia was not observed in non-pregnant NHPs, even though immune responses were similar, including the level of neutralizing antibodies [72]. The infected fetus and/or placenta has been hypothesized to act as a reservoir that cannot be efficiently cleared by the immune response and contribute to the persistent maternal ZIKV viremia observed during pregnancy; however, more studies are needed to test this hypothesis.

The paucity of clinical signs observed in infected NHP dams correlates with the observation that many ZIKV infections in humans are asymptomatic. Although most human studies in pregnant women have focused on symptomatic cases, the frequency of CZS in asymptomatic cases may be similar, making fetal demise and miscarriages from ZIKV infection more common than initially thought [78]. Among a cohort of ZIKV infections in the United States, birth defects were present in 5 and 4% of women who were symptomatic and asymptomatic, respectively [10]. This observation was recapitulated in NHPs when a combined analysis of all ZIKV pregnancy NHP studies revealed that fetal abnormalities and CZS were observed in NHP dams showing few, if any, clinical signs of disease [79]. Together, observations in NHPs and humans indicate that fetal abnormalities caused by ZIKV infection can occur regardless of maternal symptoms.

### 4.2. Fetal Infection and CZS in NHPs

Although many of the initial NHP studies reported few maternal clinical signs, ZIKV crossed the placental barrier and reached the fetus, resulting in minor brain abnormalities (Table 2). ZIKV RNA was detected in different fetal organs of rhesus and pigtail macaques, including the brain, lymph node, pericardium, and lung [72,73]. Magnetic resonance imaging scans in pigtail macaques inoculated with ZIKV showed that periventricular lesions developed in the occipital-parietal lobes of fetal brains 10 days after infection [70]. Injury to the ependyma with underlying gliosis in the posterior lateral ventricles and apoptosis of neuroprogenitor cells was also observed in the brains of infected pigtail and rhesus macaques [75,80]. This was consistent with previous studies in mice and humans showing that ZIKV infects and causes the abnormal proliferation and migration of neuroprogenitor cells [19,31,81,82,83]. When ZIKV was directly inoculated into the amniotic fluid of rhesus macaque fetuses at gestational day (GD) 50, GD64, or GD90, all fetuses exhibited periventricular and subventricular loss of neural precursors near term [84].

Infection studies in pregnant NHPs have also shown that exposure to ZIKV at earlier gestational times results in more severe outcomes for the fetus. The percentage of fetal death in utero was higher in rhesus macaques inoculated in the first trimester with a Brazilian ZIKV isolate, whereas second and third trimester infections survived to term [73]. Pregnant rhesus macaques infected with a French Polynesian ZIKV strain during the first but not third trimester resulted in fetuses that developed inflammatory ocular pathology in the retina, choroid, and optic nerve [72].

CZS was observed in NHPs when fetuses from rhesus macaque dams were inoculated with ZIKV early during gestation (<7 weeks). Fetuses infected early in pregnancy showed a reduction in occipitofrontal diameter and head circumference at term compared to non-infected NHPs, but no gross microcephaly was observed. However, these fetuses showed evidence of microcalcifications, hemorrhage, and neuronal necrosis. Fetuses that were exposed to ZIKV earlier in pregnancy also developed more severe spinal cord, ocular, and neuromuscular pathology. In comparison, fetuses exposed to ZIKV at a later time point (>12 weeks) sustained fewer neuropathologic lesions [75].

Fetal demise was also seen in NHPs infected with ZIKV. Pregnant common marmosets inoculated with a Brazilian ZIKV isolate transmitted the virus to their fetuses, which developed neuropathology and early demise [77]. In a meta-analysis of ZIKV infected pregnant NHPs at six National Primate Research Centers, fetal death was observed in 13 of 50 animals (26.0%) and early neonatal death in three animals [79]. No fetal or neonatal death was observed in macaques inoculated after GD55, whereas macaques inoculated on or before the first trimester had a rate of fetal demise of 37.8%. All cases of fetal death occurred in dams that had prolonged viremia. ZIKV RNA was detected in the amniotic and chorionic fluid, placenta, and fetus at the time of demise in most pigtail and rhesus macaques and marmosets [73,77,80].

The placenta was a target of ZIKV infection in rhesus macaques with significant pathology observed, including chorioamnionitis and deciduitis that resulted in damage to the placental villi [72,85,86]. Damage to the placenta could increase the chances of fetal abnormalities or demise following ZIKV infection. This includes a lack of oxygen transport across the villi to the fetus, as well as an increase in maternal cellular infiltration and increase in Hofbauer cells (placental macrophages) that can damage the villous architecture and alter the placenta immunological barrier [86,87]. It is also hypothesized that ZIKV may use Hofbauer cells as one of its initial targets of infection, which could enhance transmission to the fetus [87].

### 4.3. Countermeasure Studies in NHPs

The NHP model shares many aspects of human ZIKV disease during pregnancy and is important because human efficacy trials for vaccines and therapeutics may be challenging due to the waning of cases in endemic regions. NHP vaccine studies during pregnancy also are important, as highlighted by one study that showed efficacy in mice against vertical transmission, but not in pregnant rhesus macaques [27].

In the sole study to date of an antibody-based therapeutic against ZIKV infection in pregnant NHPs, a cocktail of three LALA mutant anti-ZIKV monoclonal antibodies (SMZAb1, SMZAb2, SMZAb5) was given to pregnant rhesus macaques intravenously three days post infection, at the time of peak viremia [73]. Pregnant animals were inoculated subcutaneously with a Brazilian ZIKV isolate during the third trimester (GD: 106, 125, or 134). Although viremia was controlled in two of three macaques, the virus reached the amniotic fluid in one of the animals even though viremia was controlled. In comparison, this antibody cocktail controlled viremia in non-pregnant animals, albeit when administered one day before challenge with ZIKV [88]. The authors concluded that mAb treatment following ZIKV infection in pregnant subjects is unlikely to prevent transfer of the virus to the fetus.

## 5. Other Models

Whereas mice and NHPs are most commonly used to study ZIKV infection during pregnancy, chicken eggs and pigs also have been utilized to assess the link to fetal abnormalities. Embryonated chicken eggs were used to evaluate ZIKV infection in developing embryos. Eggs injected through the amniotic membrane into the inner member space with ZIKV at E2 or E5.5 resulted in malformed chick brains [89]. High dose infections resulted in embryo loss, and ZIKV RNA was isolated from the brain, eyes, liver, heart, and crop intestine of chicken embryos.

Domestic pigs have been used to study human viral infections. Pigs have similar anatomy, genetics, and physiology to humans, although their placental structures differ [90]. Pregnant domestic pigs were inoculated with ZIKV in utero, either via intracerebral or intraperitoneal + intraaminotic routes at GD50. Viral RNA was detected in the placenta and amniotic fluid, and piglets were smaller in size at birth and developed seizures and paralysis [91]. In a separate study, ZIKV was unable to cross the porcine placenta after infection through a subcutaneous route, even though the placenta became infected when direct in utero infection was performed [92]. When the placenta was bypassed, similar results in piglets were seen, including ZIKV infection of the brain, depletion of neurons in the cerebral cortex, and microcephaly in a subset of cases.

## 6. Conclusions

In just a few years following the ZIKV outbreak in the Americas, animal models have been developed and used to study the detrimental effects of ZIKV on the developing fetus. These models have confirmed vertical transmission, the teratogenicity of ZIKV, and the ability to cause CZS. Animal models have also provided a platform for testing therapeutics and vaccines against ZIKV during pregnancy. Both NHPs and mice have shown vertical transmission after ZIKV infection, with viral RNA present in the placenta and fetal head. Fetal abnormalities that mimic CZS in humans and damage to neuroprogenitor cells have been observed in both models as well. ZIKV infection in mice and NHPs has confirmed the increased incidence of severe outcomes when infection occurs at early gestational stages. Overall, these models recapitulate many of the phenotypes seen during ZIKV infection of pregnant humans.

Despite the many advances in the field, key questions remain that these models may help to answer: (a) what is the basis for persistent viremia during pregnancy in macaques and humans?; (b) what is the role and cell type specificity of type I IFN signaling on fetal resorption?; (c) what are the long term effects of asymptomatic versus symptomatic infection during pregnancy on the mother (e.g., fertility) and children (e.g., neurodevelopmental sequelae)?; and (d) what is the significance of breakthrough infection of the fetus and placenta after vaccination? The marked waning of human infections over the past year is likely to make it challenging to perform human clinical trials and obtain new clinical specimens. Thus, small and large animal models of ZIKV infection during pregnancy will continue to have utility in addressing these and other questions, as the threat of ZIKV outbreaks and associated CZS is likely to remain in endemic regions for the foreseeable future.

## Figures and Tables

**Table 1:** Mouse Models of ZIKV Infection during Pregnancy.

Dam	Sire	ZIKV Strain(s)	Route of Infection(s)	Phenotypes	References
C57BL/6 + anti-Ifnar1 Ab	C57BL/6	H/PF/2013 (Genbank: KJ776791.2)	SC	IUGR	[19,24]
C57BL/6 + anti-Ifnar1 Ab	C57BL/6	Mouse-adapted Dakar 41525 (Genbank: MG758786)	SC or IVAG	Placental injury, viral RNA in placenta/fetal heads	[25,26,27]
*Ifnar1* ^−/−^	C57BL/6	H/PF/2013 (Genbank: KJ776791.2)	SC	Fetal resorption, IUGR, pallor, placental injury	[19]
*Ifnar1* ^−/−^	C57BL/6	FSS13025 (Genbank: KU955593)	IVAG	Fetal resorption, IUGR	[23]
C57BL/6	C57BL/6	FSS13025 (Genbank: KU955593)	IVAG	IUGR	[23]
C57BL/6	C57BL/6	SZ01 (Genbank: KU866423)	IP/Intra-ventricular	Brain malformations	[28]
C57BL/6	C57BL/6	MEX1-44 or MR-766 (Genbank: DQ859059)	Lateral ventricle of fetal brain	Reduced cortical neural progenitor cells, brain malformations, microcephaly	[29,30]
C57BL/6	C57BL/6	Brazilian clinical isolate	IV	No clinical signs in pups, no virus detected	[31]
SJL	SJL	Brazilian clinical isolate	IV	IUGR, brain malformations, ocular abnormalities	[31]
ICR	ICR	SZ01 (Genbank: KU866423)	Lateral ventricle of fetal brain	Reduced brain size, brain malformations	[32,33]
*Irf3* ^−/−^ *Irf7* ^−/−^	*Irf3* ^−/−^ *Irf7* ^−/−^	FSS13025 (Genbank: KU955593)	IVAG	IUGR	[23]
AG129	AG129	Malaysia P6-740 (Genbank: KX377336)	SC	Viral RNA in placenta and fetal heads, reduced weight and head length	[34]
C57BL/6	C57BL/6	PRVABC59 (Genbank: KU501215)	IV	Fetal resorption, little to no viral RNA in placenta and fetal heads	[35]
CD1	CD1	FSS13025 (Genbank: KU955593), Brazil Paraíba 2015 (Genbank: KX280026), PRVABC59 (Genbank: KU501215), 1968 Nigeria (Genbank: HQ234500)	IU	Viral RNA in uterine horn, placenta, fetal head, increase in number of aborted fetuses	[36]
CD1	CD1	1968 Nigeria (Genbank: HQ234500)	IP	No viral RNA in placenta or fetal heads	[36]
hSTAT2 KI	hSTAT2 KI or C57BL/6	Mouse-adapted Dakar 41525 (Genbank: MG758786) and Parent Dakar 41525 (Genbank: MG758785)	SC	Viral RNA in placenta and fetal heads	[37]
SJL	SJL	PA 259459	IV	Viral RNA in fetal heads, Sofosbuvir protected fetuses	[38]
C57BL/6 or FVB/NJ	C57BL/6 or FVB/NJ	HS-2015-BA-01 (Bahia, Brazil) (Genbank: KX520666)	IV (External jugular)	Fetal and maternal viral RNA; fetal pathology including pericardial edema and abnormal fore and hind limbs	[39]
*Rag*1^−/−^ + anti-Ifnar1 Ab	*Rag*1^−/−^ (+/− anti-Ifnar1 Ab	Brazil Paraíba 2015 (Genbank: KX280026)	IP	Vertical transmission, viral RNA in fetus, placenta, maternal tissues	[40]
*Ifnar1* ^−/−^	*Rag*1^−/−^ (+/− anti-Ifnar1 Ab	Brazil Paraíba 2015 (Genbank: KX280026)	IP	Sexual transmission (male-to-female), viral RNA in placenta and uterus	[40]
AG129	AG129	PRVABC59 (Genbank: KU501215)	SC, IVAG, Sexual	Infectious ZIKV in maternal brain, uterus, ovary, and fetus through all route of infection	[41]
C57BL/6	C57BL/6	SMGC-1 (Genbank: KX266255)	IU (via laparotomy)	Ocular pathology, microcephaly, neuronal loss, hind limb paralysis, spontaneous abortions	[42]
Swiss-Webster + anti-Ifnar1 Ab	Swiss-Webster	HN16 (Genbank: KY328289)	SC	Growth restriction in pups, decreased brain weight, viral RNA in brain and spinal cord of fetuses	[43]
ICR	ICR	GZ01 (Genbank: KU820898), FSS13025 (Genbank: KU955593)	Fetus and pup intra-ventricular	ZIKV induced microcephaly, loss of neurons, neutralizing antibody protected pups from microcephaly	[44,45]
*Ifnar1* ^−/−^	C57BL/6	Brazil Paraíba 2015 (Genbank: KX280026)	SC	Fetal resorption, viral RNA in fetus and placenta, microcephaly	[46]
C57BL/6 + anti-Ifnar1 Ab	C57BL/6	2015 Paraíba (Genbank: KX280026)	SC	Viral RNA in placenta (decidua and junctional zone) and fetal heads, maternal brain, spleen	[46]
*Ifnlr1*^−/−^ + anti-Ifnar1 Ab	C57BL/6 or *Ifnlr1*^−/−^	2015 Paraíba (Genbank: KX280026)	SC	Viral RNA in placenta (decidua and junctional zone) and fetal heads, maternal brain, spleen	[46]
C57BL/6	C57BL/6	SZ01 (Genbank: KU866423)	IA	Decrease in brain volume and cortical thickness of fetuses, paralysis and motor function deficits in pups	[47]
C57BL/6	C57BL/6	ZIKA-SMGC-1 (Genbank KX266255)	IU	Pups developed microcephaly, ocular defects, paralysis	[42]
C57BL/6 (H-2^b^) + anti-Ifnar1 Ab	Balb/c (H-2^d^)	PRVABC59 (Genbank: KU501215) and MR766 (Genbank: DQ859059)	SC	Viral RNA in fetal tissue and maternal serum, spleen, liver, and brain	[48]
Balb/c + anti-Ifnar1 Ab	Balb/c	GZ01 (Genbank: KU820898) and FSS13025 (Genbank: KU955593)	SC	Viral RNA in fetal head and placenta, hemorrhagic uterus	[49]
*Ifnar1*^−/−^*Ifngr1*^−/−^ (AG6)	*Ifnar1*^−/−^*Ifngr1*^−/−^ (AG6)	SZ-WIV01 (KU963796)	IP	*In utero* death	[50]
*Ifnar1* ^−/−^	C57BL/6	Brazil Paraiba 2015 (Genbank: KX280026)	SC	Fetal resorption	[25,26]
*Ifnar1* ^−/−^	C57BL/6	FSS13025 (Genbank: KU955593)	SC or IV	Fetal resorption, decreased fetal size	[51]
C57BL/6 + anti-Ifnar1 Ab	C57BL/6	FSS13025 (Genbank: KU955593)	IV	Viral RNA in fetal tissue, placenta, maternal serum, brain, and spleen	[51]
Atg161^HM^ + anti-Ifnar1 Ab	Atg161^HM^	Brazil Paraiba 2015 (Genbank: KX280026)	SC	Reduction in viral RNA in placenta and fetus compared to WT controls	[52]
Cyp 19 Cre^+^ Atg16L1^fl/fl^ + anti-Ifnar1 Ab	Cyp 19 Cre^+^ Atg16L1^fl/fl^	Brazil Paraiba 2015 (Genbank: KX280026)	SC	Restricted ZIKV infection in placenta compared to controls	[52]
A129	A129	PRVABC59 (Genbank: KU501215)	SC	Viral RNA in placenta, fetal head, maternal brain, reduced fetal weight	[53]

SC = Subcutaneous; IP = Intraperitoneal; IVAG = Intravaginal; IV = Intravenous; IU = Intrauterine; IA = Intraaminotic; IUGR = intrauterine growth restriction.

**Table 2:** Non-Human Primate Models of ZIKV Infection during Pregnancy.

Model	ZIKV Strain	Route	Dose	Time Infected	Number NHPs Infected	Phenotypes	References
Rhesus macaque	H/PF/2013 (GenBank: KJ776791.2)	SC	10^4^ PFU	GD: 31 or 38	2	Persistent viremia	[71]
Rhesus macaque	H/PF/2013 (GenBank: KJ776791.2)	SC	10^4^ PFU	GD: 31, 38, 104, or 199	4	Persistent viremia, viral RNA in fetus	[72]
Rhesus macaque	PRVABC59 (Genbank: KU501215.1)	SC	10^5^ FFU	GD: 31, 51, 114, or 115	5	Viral RNA in fetus, placenta damage, decreased oxygen permeability, villous calcification, stromal proliferation, fibrin deposition, subtle fetal brain abnormalities in 2 of 5 animals (second and third trimester)	[86]
Rhesus macaque	Brazil ZKV2015 (GenBank: KU497555.1)	SC	10^3^ PFU	GD: 42–98	9	Persistent viremia, fetal neuropathology, fetal loss, smaller brain size, brain lesions, apoptosis of neuroprogenitor cells, placental pathology	[75]
Rhesus macaque	PRVABC59 (Genbank: KU501215.1)-Barcoded	SC	10^4^ PFU	GD: 35	1	Persistent viremia	[76]
Rhesus macaque	Rio U-1/2016 (Genbank: KU926309)	SC	10^4^ PFU	GD: 44–124	11	Virus in the amniotic fluid (AF); and in utero fetal deaths	[73]
Rhesus macaque	Brazil SPH2015 (GenBank: KU321639.1	IA	10^5^ PFU	GD: 41, 50, 64, or 90	4	Fetal demise, high viral RNA levels in fetal and placental tissues, including brain; fetuses carried to near term and euthanized displayed no gross microcephaly, but had brain calcifications and reduced neural precursor cells	[84]
Pigtail macaque	Cambodia FSS13025 (GenBank: KU955593.1)	SC	5 × 10^7^ PFU	GD: 119	1	Ultrasound lag in growth biparietal diameter, fetal brain lesions, viral RNA in fetal brain, liver, placenta	[70]
Pigtail macaque	Cambodia FSS13025 (GenBank: KU955593.1)	SC	5 × 10^7^ PFU	GD: 82 or 119 ^3^	2	Ependymal injury in posterior lateral ventricles, fused ventricular surfaces, periventricular gliosis, white matter GFAP+ gliosis, axonal spheroids, deficient fetal neuronal progenitor cells, loss of non-cortical volume in fetal brain (silent pathology)	[80]
Pigtail macaque	Brazil Fortaleza 2015 (GenBank: KX811222.1)	SC ^1^	5 × 10^7^ PFU	GD: 60–63	3	Ependymal injury in posterior lateral ventricles, fused ventricular surfaces, periventricular gliosis, white matter GFAP+ gliosis, axonal spheroids, deficient fetal neuronal progenitor cells, loss of non-cortical volume in fetal brain (silent pathology)	[80]
Marmoset	Brazil SPH2015 (GenBank: KU321639.1)	IM ^2^	2.5 × 10^5^ PFU	GD: 68 or 79	2	Fetal demise, abortion, viral RNA in placenta and fetus	[77]

SC = Subcutaneous; IA = Intraamniotic; IM = Intramuscular; GD: Gestational day. ^1^ Salivary gland extract/monoclonal antibody given before and after infection. ^2^ Second inoculation four days later. ^3^ 119 gestation NHP was same one in 2016 Adams Waldorf et al. publication.
